# Estimation of Height at Withers Based on Long Bone Measurements of Living Cats

**DOI:** 10.3390/vetsci11110522

**Published:** 2024-10-28

**Authors:** Dominik Poradowski, Zihni Mutlu, Yusuf Altundağ, Aleksander Chrószcz, Özlem Sarıtaş, Joanna Wolińska, Vedat Onar

**Affiliations:** 1 Department of Biostructure and Animal Physiology, Faculty of Veterinary Medicine, Wrocław University of Environmental and Life Sciences, ul. Kożuchowska 1, 51-631 Wroclaw, Poland; 2 Department of Surgery, Faculty of Veterinary Medicine, Istanbul University-Cerrahpaşa, 3420 Avcılar, Türkiye; 3 Department of Prehistory, Division of Archaeology, Faculty of Arts and Sciences, Hitit University, 19030 Çorum, Türkiye; 4 Faculty of Veterinary Medicine, Wrocław University of Environmental and Life Sciences, ul. C.K. Norwida 31, 50-375 Wrocław, Poland; 5 Osteoarchaeology Practice and Research Centre & Department of Anatomy, Faculty of Veterinary Medicine, Istanbul University-Cerrahpaşa, 34320 Avcılar, Türkiye

**Keywords:** cat, height at withers, long bones, slenderness, gender

## Abstract

In this study, coefficients enabling estimation of shoulder height were obtained by taking morphometric measurements from radiographic images of live cats using the radiogrammetric method. For this purpose, a total of 37 adult cats, including 17 females and 20 males, were involved. While the effect of sexual dimorphism was observed on the morphometric data, the presence of sexual dimorphism was only evident in the coefficients (factors) of the humerus and talus. No significant effect of gender was found on the shoulder height estimation coefficients (factors) for other thoracic and pelvic limb bones. Gender had an impact on the slenderness index in all thoracic limbs, but only on the tibia slenderness index among the pelvic limb bones. Generally, female individuals were found to have more slender limbs. It is believed that the obtained coefficients (factors) and index data will contribute significantly to the prediction of archaeological cat morphology.

## Simple Summary

The study deals with new method of the shoulder height estimation in cats using the measurements available in the x-ray examination. The imaging of limbs skeleton in 37 domestic cats (17 queens and 20 tomcats) were used for the mathematic equation development, which allows for computing the shoulder height on the basis of the greatest length of subsequent limb’s bones. The sexual differences were found only in the humerus and talus. The gender influenced also the slenderness of the bones. The achieved results proved that the new method is more accurate than used routinely earlier and introduced by Koudelka over 100 years ago. The values of the shoulder height computed with the new method were also closer to the values measured using the zoometric stick (the tool used in shoulder height estimation in standing animal).

## 1. Introduction

Body size is a crucial factor in animal ecology, particularly in terrestrial animals, where mechanical properties of the skeleton play a significant role [1]. Therefore, the body size is of great importance for the morphological appearance of animals, and exerts a noticeable impact on the life history of an organism [2,3].

Differences in body mass are reflected in the thoracic limbs and pelvic limbs, which form the skeletal suspension system of body weight. The length of appendicular bones affects the height at the withers and the length of the extremities. Bone length and width reflect limb slenderness. However, it is reported that slenderness of the limbs and height at the withers or rump can be associated with phenotypic or environmental differences [4,5]. The slenderness (shape) index is considered an indicator of the robustness of a specific element. When compared with withers height/shoulder height, an individual’s slenderness index helps determine whether the animal has limbs that are proportionally thinner or sturdier [6].

Various scientists have employed different formulations based on long bone measurements to predict body mass, that is, an indicator of body size, for various animal species [1,7,8,9,10,11,12,13]. Another indicator associated with body size is shoulder or scapula height. Therefore, predictions of shoulder or withers height with morphometric data from long bones are commonly used in zooarchaeological studies [14,15,16,17,18,19,20,21,22]. They allow for interpretations regarding body mass and visual morphological characteristics based on shoulder or withers height. Additionally, shoulder/withers height is considered an important morphological factor in describing animal populations. This is because it facilitates direct comparisons of measurements of different elements and helps visualize a live animal without measuring its bone length [23].

Although there are various methods for predicting the visible morphological shape and shoulder height in domestic animals, many of them have been suggested to be impractical for zooarchaeologists. Nevertheless, the most common methods are still using the morphometric data from long bones [14,15,16,17,18,19,20,21,22]. In the predictions of shoulder height, the greatest length (GL) of long bones is typically used as a variable, and regression equations are constructed based on this parameter. Such regression equations or coefficients have been established for horses, donkeys, sheep, goats, cattle, pigs, dogs, and deer [14,15,16,17,18,19,22]. However, as cats are important pets for humans, a formulation for predicting shoulder height in cats was coined over 100 years ago. The Koudelka method for estimating cat height at the withers is still used in archaeozoological studies, even though new imaging techniques have been incorporated in veterinary sciences.

In this study, morphometric measurements were taken from radiographic images of feline long bones using the non-invasive radiogrammetric method. The relationship between these measurements and shoulder height was evaluated. Our aim was to enable the estimation of shoulder height in cats based on long bone measurements and, consequently, to provide interpretation of visual morphological features. Additionally, the presence of sexual dimorphism in long bone measurements was investigated, to highlight changes and differences in predicting shoulder height based on gender. The results achieved with both classical osteometric and radiogrammetric method were comparable.

## 2. Materials and Methods

In this study, a total of 37 adult cats were examined, including 17 females and 20 males. All animals were ordinary patients of the Department of Surgery, Faculty of Veterinary Medicine, Istanbul University-Cerrahpaşa, and manifested in clinical examination the symptoms of diseases not indicating any pathologies influencing the locomotor system morphology. Bone and joint diseases were excluded. All animals were kept in human indoor households. During clinical examination, all were diagnosed as healthy (prior vaccinations). The animals were adult (3 to 5 y. o.). All animals’ owners consented to the use of their animals’ X-rays in this study. After obtaining data on the age, gender, and shoulder height of the cats, radiographs were taken to cover all the bones of their left thoracic and pelvic limbs.

Single-direction (left) imaging aimed to minimize the exposure of the cats to X-rays. The direction was determined randomly. Non-invasive radiographic imaging was performed on the cats; the radiographs were taken after pharmacological immobilisation routinely used in the veterinary practice.

**Figure 1:**
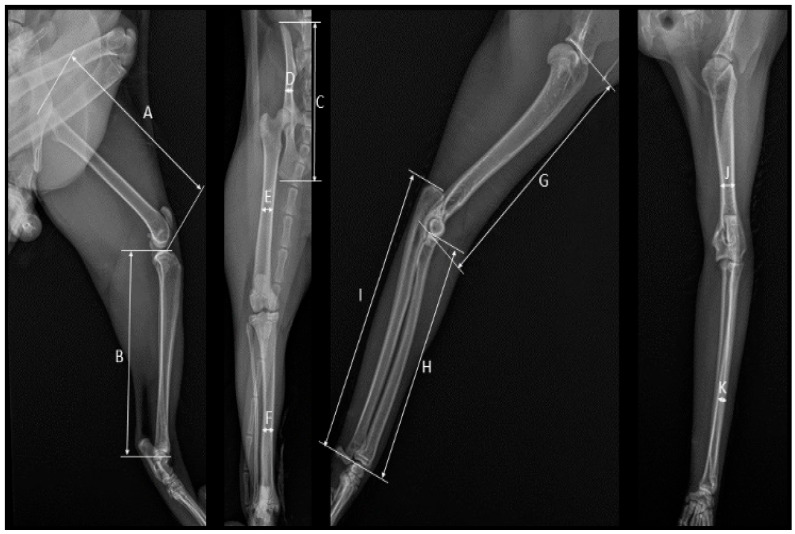
Radiographic imaging of feline thoracic and pelvic limbs. A. Femur greatest length (GL); B. Tibia greatest length (GL); C. *Os coxae* greatest length (GL); D. *Os coxae* smallest breadth of the shaft of ilium (SD); E. Femur smallest breadth of diaphysis (SD); F. Tibia smallest breadth of diaphysis (SD); G. Humerus greatest length (GL); H. Radius greatest length (GL); I. Ulna greatest length (GL); J. Humerus smallest breadth of diaphysis (SD); K. Radius smallest breadth of diaphysis (SD).

Two-directional radiographs of the left limbs were taken in dorsopalmar/dorsoplantar and mediolateral projections (Figure 1). Imaging was performed based on the shooting method called metacarpal radiogrammetry [24]. To ensure the most accurate imaging of the long bones, a cassette was kept close to the thoracic and pelvic extremities, with the horizontal angle not exceeding 5 degrees [22,25], and care was taken to shoot from a maximum of 1 metre with an optimal focus distance of up to 90 degrees. This ensured accuracy and standardization of the measurements taken from the obtained images [25].

The cats used in the study were selected as adult individuals without bone pathologies. After transferring the images to the computer, morphometric measurements were taken from the images, primarily based on the von den Driesch method [26].

Morphometric measurements [26].

For the humerus, radius, ulna, femur, tibia, talus, and calcaneus:

1. Greatest length (GL)
2. Smallest breadth of diaphysis (SD)

For the os coxae:

1. Greatest length of *os coxae* (GL)
2. Smallest breadth of the shaft of ilium (SD)

Using these morphometric measurements, we calculated the slenderness index [6,27,28,29], with the following formula:

Slenderness index = Smallest breadth of diaphysis (SD)* 100/Greatest length (GL)

After completing all the imaging and measurements, statistical analyses were conducted using the SPSS 21.0 package (Version 21.0, SPSS Inc., Chicago, IL, USA). First, we examined the effect of gender on the long bone measurements, and then we attempted to demonstrate the statistical impact of sexual dimorphism. Applied statistical analyses included average value (mean), standard deviation, median, and Student t-test (to evaluate the difference between genders). The regression analysis was performed (dependent = shoulder height, independent = morphometric value (GL)). In this analysis, the R2 value was taken into consideration as the determinant value. Since much zooarchaeological literature uses the ‘direct shoulder height/GL ratio’ calculation instead of the regression formula, this calculation was also used in our study.

To estimate shoulder height in cats using morphometric measurements, a direct shoulder height/GL ratio was calculated. Due to low R^2^ (determinacy) values of the regression formulas, a factor calculation method widely used in zooarchaeology [14,15,16,17,18,19] was adopted in this study. Thus, the factors obtained for cats contributed to the estimation of shoulder height and to the prediction of visual morphology in both zooarchaeology and forensic veterinary science. As Koudelka [14] had already estimated the factors for the mathematical calculations of animal height at the withers over 100 years ago, we tried to compare the accuracy of this method with ours.

## 3. Results

In the cats examined in the study, we demonstrated the influence of sexual dimorphism on shoulder height. Specifically, the shoulder height of male individuals was greater than that of females (Table 1).

Morphometric data for the thoracic and pelvic limb bones, as well as the indices and factor values obtained with them, are presented in Table 2 and Table 3. The influence of gender was observed for the GL and SD values of the humerus, radius, and ulna. The values for these bones were greater in males than females, and the difference was significant at *p* < 0.05.

Gender effects were also observed in the slenderness index for the humerus and radius. Female individuals had thinner bones and, consequently, thoracic limbs as compared with males. A slenderness index calculation for the ulna could not be performed as the SD value was not measurable.

The factors obtained from the humerus, radius, and ulna, which were used in predicting shoulder height, are provided in Table 2. The influence of gender on these factors was evident, with mean differences between female and male individuals not significant at *p* > 0.05.

The morphometric data for the pelvic limb bones are presented in Table 3. As with the thoracic limbs, gender dimorphism was noticed for the pelvic limb skeleton. However, while sexual dimorphism was observed in the GL value of the *os coxae*, there was no gender effect on the smallest breadth of the shaft of the ilium.

The influence of gender on the slenderness index was observed only in the tibia, with average differences between male and female individuals significant at *p* < 0.05. The female individuals had thinner tibias.

We found no significant differences in the average slenderness index for the *os coxae* and femur between the female and male individuals. Therefore, sexual dimorphism in these bones was not observed based on the slenderness index.

As far as the pelvic limb bones were concerned, we found no gender-related differences except for the talus. A significant difference in the mean factor values for the talus was found between the female and male individuals at *p* < 0.05.

The height at the withers, assessed with the Koudelka method [14] and computed based on radiogrammetry, is compared in Table 4. The statistical analysis proved that the accuracy of the radiogrammetric method showed better correlation with the known height at the withers than in the Koudelka method (*p* < 0.05). Moreover, the results for the other long bones showed no statistically different values. The values computed using the Koudelka factors were higher and less comparable with the known shoulder height in the examined cats.

Due to the fact that the median is a less influenced value than the mean, it was calculated for withers/shoulder height calculations in both tested methods (Table 5).

The median is more resistant for extremal simple values and shows the differences between both methods more objectively. The median and mean values calculated for all samples were the same, while differences between them were seen in the male and female groups. The median computed for the Koudelka method results was higher than for the radiogrammetrically estimated values. The difference was more visible in the male than in the female group. These results proved that the radiogrammetric method gives values more comparable with known animal height.

## 4. Discussion

Archaeozoologists focus on the importance of morphological data in defining animal populations [30]. Attempts are made to predict shoulder height by considering the close relationship between the limb bone length and the height at the withers. Moreover, the accessible literature describes mathematical formulas for the shoulder height estimation based on skull measurements in dogs, roe deer, and horses [15,31,32,33]. Even though these methods are characterised by similar accuracy to the long bone morphometric results, finding well-preserved cranial skeletons (skulls) is rare. Shoulder height, or withers height, is considered a significant morphological factor in describing animal populations. This is because it incorporates measurements of different elements (long bones) and allows for a direct comparison of measurements of various elements [30]. One of the most important methods in this area involves morphometric measurements of long bones [14,30].

The withers or shoulder height, an indicator of body size, is generally estimated by various scientists based on different formulas or factors using archaeological animal long bone measurements. These formulas or factors, which vary according to animal species, are still widely valid estimation methods today [14,15,16,17,19,34,35,36,37,38,39]. However, it is a known fact that specific formulas or factors for the same species do not yield similar results when applied to archaeological animal bones [40,41].

While shoulder height estimation is performed in a wide range of animal populations from horses to cattle, sheep, or dogs, its prediction in pet animals has been limited to dogs only [38]. These methods of height at withers estimation have been partly verified by other authors [23,32,33,42]. The well-known Koudelka method of shoulder height estimation, based on long bone osteometry, has never been revised or compared with modern techniques of imaging. It is based on classical osteometry with the use of slide-callipers for GL measurement, which is then used in a mathematical formula with a coefficient estimated by the author for each long bone. In this study, we present data that contribute to predicting visual morphology from common archaeological cat remains. Similar to Koudelka [14], the radiogrammetric method is based on the coefficient calculation for each long bone’s GL, which can be used in the withers/shoulder height estimation. Both methods’ results comparison (Table 4) proved that the radiogrammetric method achieved values more comparable with known animal withers/shoulder height, due to better correlation between them, and the Koudelka results were higher than those of a computer on the basis of X-ray measurements. These findings allowed for critical revision of only one accessible classical method of withers/shoulder height estimation in cats.

The cats used in this study were kept indoors and diagnosed as healthy (prior vaccinations). They belonged to the European shorthaired cat type, which seems to be the best for use in zooarchaeological analyses due to the lack of advanced breeding influence on the morphotype. The chosen individuals were not showing any signs of being overweight or obese. This experimental group seems to resemble more the early domestic cats from the past than other breeds, allowing for the estimation of coefficients more adequately.

The coefficient of determination (R^2^) obtained from the regression calculations was not sufficiently high, and even when these formulas included both GL and SD, the percentage did not exceed 40%. Such low values of the coefficient were not considered desirable in terms of determination. Therefore, in our study, factors (coefficients) were obtained using the ratio of shoulder height (kg) to GL value (cm), a common method still used in zooarchaeology [14,15,16,17,18,19,36,38]. Although the effect of sexual dimorphism was observed in the morphometric data, determining gender based on long bones is a considerable challenge. Still, gender-related factors should be considered when predicting shoulder height for total archaeological cat skeletons, whose gender is estimated by different methods (e.g., DNA analysis). However, the gender-related factors were statistically insignificant for the average values measured for the other bones, except for the humerus and the talus.

Body mass and differences in visual morphology are crucial considerations due to their impact on weight-bearing thoracic and pelvic limbs, making the slenderness index highly important. When compared with the withers height, the individual slenderness index scaled to the animal height allows for determining whether the limbs are thinner or sturdier [6].

While the slenderness index is commonly used in ruminants and horses [6,27,28,29], there is a lack of data on its use in other species. This study presents the slenderness index values for the thoracic and pelvic limb bones in cats. Female individuals generally have a thinner limb skeleton, and the effect of sexual dimorphism is only observed in the tibia slenderness index.

## 5. Conclusions

In conclusion, the estimation of visual morphology, especially of shoulder height, from archaeological cat remains has not been addressed until now. The Koudelka method of shoulder height estimation in cats was introduced over 100 years ago. Today, the incorporation of more accurate and modern methods can not only verify its accuracy, but also supply much needed modifications. Our study proved that the results achieved with the Koudelka method and our formula are comparable with animal size increase, but radiogrammetry yields more accurate results than the classic methodology. Moreover, the factors we described can be easily used not only on X-rays, but also in routine archaeozoological practice. In our study, coefficients enabling estimation of the shoulder height in cats were determined using morphometric data obtained from live animals based on the radiogrammetric method. This offers the possibility of comparing shoulder height across different feline populations.

Additionally, the morphometric data for the thoracic and pelvic limbs not only facilitate the prediction of visual morphology, but also contribute to defining the morphology of both types of appendices in cat populations. This is because the predicted withers height based on osteometric measurements of limbs can provide meaningful information about the entire live animal, without the need for bone lengths, and it can also facilitate direct comparisons of measurements of different elements [30]. Therefore, it is believed that this approach is suitable for cats.

## Figures and Tables

**Table 1:** Shoulder height [cm]. n—number of individuals, mean—mean value.

Gender	Statistical	Shoulder Height
FEMALE	N	17
	mean	24.68 ± 2.4 ^a^
MALE	N	20
	mean	27.09 ± 2.21 ^b^
TOTAL	N	37
	mean	25.98 ± 2.57

^a,b^: Differences between the means indicated by different letters on the same line are significant (*p* < 0.05).

**Table 2:** Morphometric values of the thoracic limb [cm]. n—number of individuals, mean—mean value, GL—greatest length, SD—smallest breadth of the shaft of ilium/diaphysis.

Bone	Gender	Statistical Parameter	GL	SD	Slenderness Index	Factors
HUMERUS	FEMALE	n	17	17	17	17
		mean	9.68 ± 0.59 ^a^	0.75 ± 0.06 ^a^	7.76 ± 0.67 ^a^	2.55 ± 0.25 ^a^
	MALE	n	20	20	20	20
		mean	10.39 ± 0.62 ^b^	0.82 ± 0.07 ^b^	7.88 ± 0.56 ^a^	2.61 ± 0.24 ^b^
	TOTAL	n	37	37	37	37
		mean	10.06 ± 0.7	0.79 ± 0.07	7.82 ± 0.61	**2.59 ± 0.24**
RADIUS	FEMALE	n	16	16	16	16
		mean	9.32 ± 0.57 ^a^	0.49 ± 0.07 ^a^	5.29 ± 0.61 ^a^	2.65 ± 0.24 ^a^
	MALE	n	20	20	20	20
		mean	10.07 ± 0.55 ^b^	0.56 ± 0.05 ^b^	5.57 ± 0.48 ^a^	2.70 ± 0.23 ^a^
	TOTAL	n	36	36	36	36
		mean	9.74 ± 0.67	0.53 ± 0.07	5.45 ± 0.62	**2.68 ± 0.22**
ULNA	FEMALE	n	16			16
		mean	10.94 ± 0.61 ^a^	-	-	2.26 ± 0.17 ^a^
	MALE	n	20			20
		mean	11.81 ± 0.56 ^b^	-	-	2.3 ± 0.18 ^a^
	TOTAL	n	36			36
		mean	11.43 ± 0.72	-	-	**2.28 ± 0.18**

^a,b^: Differences between the means indicated by different letters on the same line are significant (*p* < 0.05).

**Table 3:** Morphometric values of the pelvic limb [cm]. n—number of individuals, mean—mean value, GL—greatest length, SD—smallest breadth of the shaft of ilium/diaphysis.

Bone	Gender	Statistical Parameter	GL	SD	Slenderness Index	Factors
COXAE	FEMALE	N	16	16	16	16
		Mean	8.06 ± 0.41 ^a^	0.57 ± 0.05 ^a^	7.06 ± 0.46 ^a^	3.07 ± 0.26 ^a^
	MALE	N	20	20	20	20
		Mean	8.73 ± 0.35 ^b^	0.61 ± 0.06 ^a^	6.96 ± 0.63 ^a^	3.11 ± 0.27 ^a^
	TOTAL	N	36	36	36	36
		mean	8.43 ± 0.5	0.59 ± 0.06	7.00 ± 0.55	**3.09 ± 0.26**
FEMUR	FEMALE	N	16	16	16	16
		mean	10.22 ± 0.62 ^a^	0.88 ± 0.07 ^a^	8.63 ± 0.74 ^a^	2.42 ± 0.27 ^a^
	MALE	N	20	20	20	20
		mean	11.27 ± 0.66^b^	0.97 ± 0.08 ^b^	8.58 ± 0.57 ^a^	2.41 ± 0.25 ^a^
	TOTAL	N	36	36	36	36
		mean	10.81 ± 0.83	0.93 ± 0.09	8.60 ± 0.64	**2.42 ± 0.25**
TIBIA	FEMALE	N	16	16	16	16
		mean	11.05 ± 0.61 ^a^	0.78 ± 0.07 ^a^	7.04 ± 0.5 ^a^	2.24 ± 0.24 ^a^
	MALE	N	20	20	20	20
		mean	11.65 ± 0.55 ^b^	0.86 ± 0.05 ^b^	7.39 ± 0.39 ^b^	2.33 ± 0.21 ^a^
	TOTAL	N	36	36	36	36
		mean	11.38 ± 0.65	0.82 ± 0.07	7.24 ± 0.47	**2.29 ± 0.22**
TALUS	FEMALE	N	15	-	-	15
		mean	1.54 ± 0.1 ^a^	-	-	15.86 ± 1.35 ^a^
	MALE	N	20	-	-	20
		mean	1.69 ± 0.06 ^b^	-	-	16.01 ± 1.37 ^b^
	TOTAL	N	35	-	-	35
		mean	1.63 ± 0.11	-	-	**15.95 ± 1.34**
CALCANEUS	FEMALE	N	17	-	-	17
		mean	2.71 ± 0.14 ^a^	-	-	9.10 ± 0.73 ^a^
	MALE	N	20	-	-	20
		mean	3.01 ± 0.1 ^b^	-	-	9.01 ± 0.71 ^a^
	TOTAL	N	37	-	-	37
		mean	2.87 ± 0.19	-	-	**9.05 ± 0.71**

^a,b^: Differences between the means indicated by different letters on the same line are significant (*p* < 0.05).

**Table 4:** Results for height at the withers calculations [cm] with the radiogrammetric method (R) and the Koudelka (K) method.

Method	Bone	Female	Male	Total
R	HUMERUS	24.68 ± 2.4	27.09 ± 2.21	25.98 ± 2.57
K		27.39 ± 1.67	29.41 ± 1.77	28.48 ± 1.98
R	RADIUS	24.72 ± 2.48	27.09 ± 2.21	2.59 ± 2.48
K		27.49 ± 1.68	29.7 ± 1.63	28.72 ± 1.97
R	ULNA	24.72 ± 2.48	27.09 ± 2.21	26.04 ± 2.59
K		26.6 ± 1.49	28.7 ± 1.36	27.76 ± 1.75
R	FEMUR	24.72 ± 2.33	27.09 ± 2.21	26.04 ± 2.59
K		26.28 ± 1.6	28.98 ± 1.7	27.78 ± 2.13
R	TIBIA	24.72 ± 2.48	27.09 ± 2.21	26.04 ± 2.59
K		27.06 ± 1.5	28.54 ± 1.35	27.88 ± 1.58

**Table 5:** The median value computed for both methods of the withers/shoulder height estimation. Radiogrammetric method (R) and Koudelka (K) method.

Method	Female	Male	Total
**R**	**25.0**	**28.0**	**26.0**
**K**	**27.39**	**29.52**	**26.0**

## Data Availability

The original contributions presented in the study are included in the article material. Further inquiries can be directed to the corresponding author.
